# Clinical Presentation and Imaging Findings of Irreversible Cerebral Amyloid Angiopathy-Related Inflammation (CAA-ri): A Case Report and Literature Review

**DOI:** 10.7759/cureus.66475

**Published:** 2024-08-08

**Authors:** Masahiro Hayashi, Kotaro Higashi, Katsuji Kobayashi

**Affiliations:** 1 Neurology and Psychiatry, Medical Corporation Asanokawa, Sakuragaoka Hospital, Kanazawa, JPN; 2 Radiology, Medical Corporation Asanokawa, Asanokawa General Hospital, Kanazawa, JPN; 3 Psychiatry, Awazu Neuropsychiatric Sanatorium, Komatsu, JPN

**Keywords:** cerebral amyloid angiopathy-related inflammation, cerebral microbleed (cmb), posterior reversible encephalopathy syndrome (pres), corticosteroids therapy, white matter lesion

## Abstract

Cerebral amyloid angiopathy-related inflammation (CAA-ri) is a rare condition primarily driven by an autoimmune reaction against cerebrovascular amyloid beta protein. Accurate diagnosis hinges on recognizing characteristic clinical symptoms and imaging features, such as asymmetric cerebral white matter lesions often linked to angioedema. We report the case of a woman in her 70s with progressive, irreversible CAA-ri who initially presented with left homonymous hemianopia and experienced significant psychiatric and neurological deterioration following an epileptic seizure. Despite initiating corticosteroid therapy seven months after onset, her condition continued to worsen, ultimately leading to her death in the 11th month due to general decline. This report reviews the clinical progression and imaging findings of the case, discusses the diagnostic process for CAA-ri, differentiates it from related conditions, and evaluates the timing of corticosteroid treatment.

## Introduction

Cerebral amyloid angiopathy-related inflammation (CAA-ri) is a rare disease primarily caused by an autoimmune reaction against the cerebrovascular amyloid beta (Aβ) protein [1,2]. As a subtype of CAA, CAA-ri is associated with elevated anti-Aβ42 antibodies in CSF during the disease’s active phase [3]. While a definitive diagnosis of CAA-ri typically requires a brain biopsy, the diagnostic criteria proposed by Auriel et al. in 2016 offer a more accurate diagnosis based on histological verification, characteristic clinical symptoms, and imaging findings [4]. CAA-ri is clinically diagnosed in individuals over 40 and is marked by acute and subacute headaches, cognitive impairment, and seizures. Brain imaging, particularly MRI, reveals focal or multifocal, often asymmetric white matter lesions (WMLs) derived from angioedema. Additionally, hemorrhagic lesions such as cerebral microbleeds (CMBs) and cortical superficial siderosis (cSS) are also characteristic findings [4].

CAA-ri is generally regarded as a reversible condition, with spontaneous remission occurring in about 14% of cases. Treatment with corticosteroids and immunosuppressants improves clinical symptoms and imaging findings in approximately 70-80% of patients [1,5,6]. However, about 40% of those who achieve remission may experience a relapse, with some developing a progressive disease course or severe complications, such as intracerebral hemorrhage [1,5,7]. Therefore, prompt and appropriate diagnosis and treatment are crucial.

In this report, we describe the case of a female patient in her 70s who initially presented with left homonymous hemianopia and WMLs in the right occipital lobe. Her condition progressed to include epileptic seizures along with various psychiatric and neurological symptoms. Approximately two years prior, she had been treated for hypopharyngeal cancer at a local general hospital and had been under follow-up there. Despite developing left homonymous hemianopia, she continued treatment at the same facility. Although a brain biopsy was considered to confirm CAA-ri, the worsening of her psychiatric symptoms, such as visual hallucinations, paranoia, and irritability, complicated the procedure. Seven months after the onset of her symptoms, the patient, whose condition also involved deteriorating motor and cognitive functions and increasing somnolence, was transferred to our hospital. Despite receiving three high-dose corticosteroid pulse therapies for CAA-ri, the response was limited. Her general condition continued to decline, and she passed away in the 11th month after the onset of her illness.

Based on the medical information and imaging data from our clinic and previous institutions, as well as the daily life information provided by the patient’s family, we will present the clinical symptoms, disease course, and imaging findings. We will discuss the diagnosis of CAA-ri, differentiate it from similar conditions, and review treatment options and their appropriate timing, supported by a literature review.

## Case presentation

We present the case of a 74-year-old right-handed woman who had previously undergone gastric subtotal resection in her 30s. Three years ago, she was diagnosed with stage III hypopharyngeal cancer and received treatment at a local general hospital for approximately three months. Her treatment included chemotherapy (cisplatin: 40 mg/m², administered intravenously five times) and radiotherapy (intensity-modulated radiotherapy: 2 Gy daily for 35 sessions). This combination led to the remission of the hypopharyngeal cancer, with no complications during treatment. After being discharged, she continued with follow-up care and lived a stable recuperative life at home with her husband.

Twenty-one months after completing treatment for hypopharyngeal cancer, the patient suddenly developed visual impairment and was urgently referred back to the same hospital, where she was diagnosed with left homonymous hemianopia. Following this diagnosis, she began experiencing various neuropsychiatric symptoms, particularly after epileptic seizures. Notable psychiatric symptoms included complex visual hallucinations in the left hemianopic field and paranoia associated with these hallucinations.

**Figure 1:**
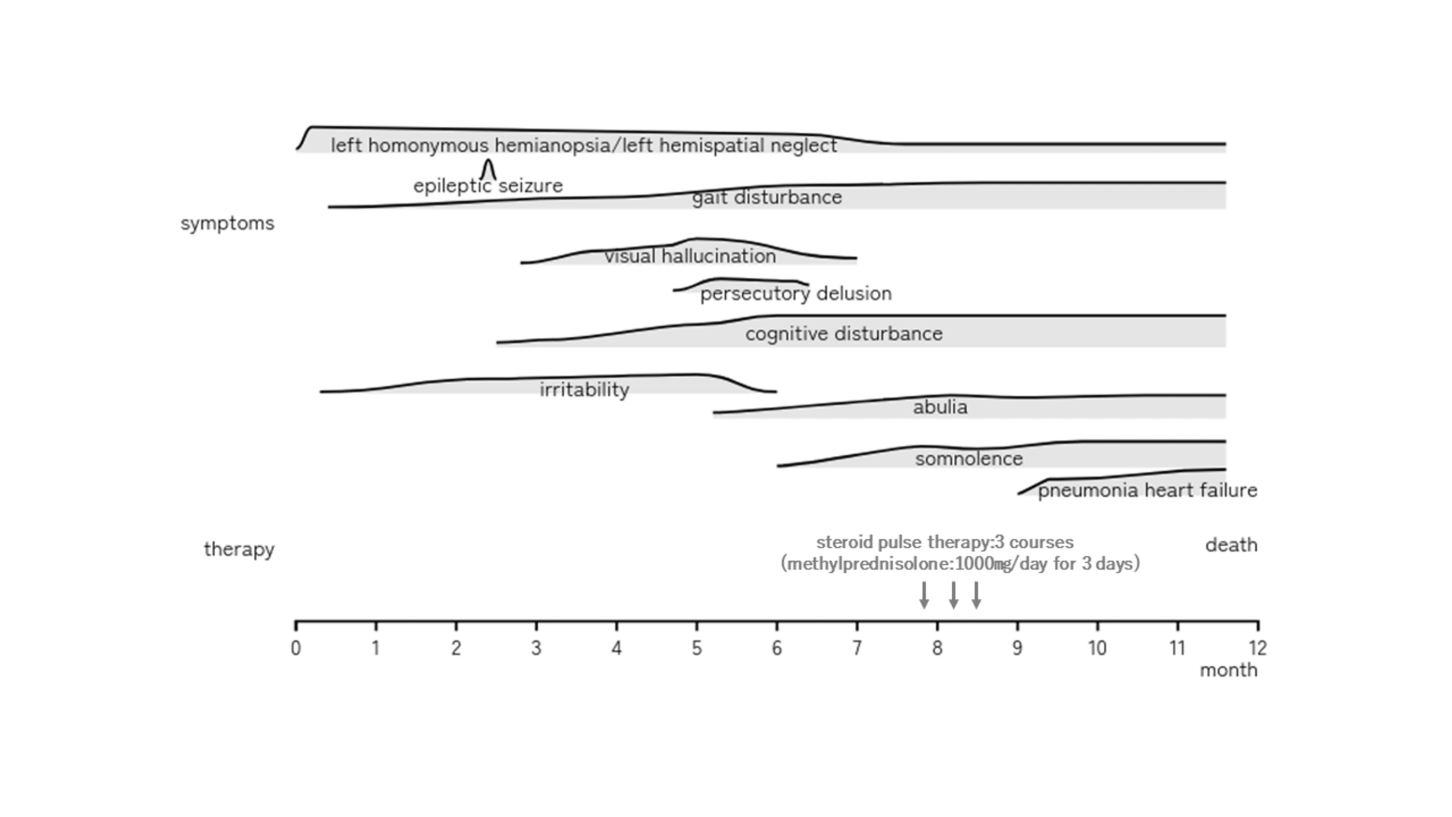
Clinical course of a female CAA-ri patient in her 70s CAA-ri, cerebral amyloid angiopathy-related inflammation

In terms of motor function, the patient developed balance issues and a worsening gait, in addition to effects related to left hemispatial neglect. Initially, her cognitive functions, including memory, orientation, and language, were preserved. However, impairments in executive function and attention emerged, with a slowly progressive course. This was followed by the onset of abulia, leading to a rapid decline in cognitive functions and severe general cognitive disturbance (Figure 1).

**Figure 2:**
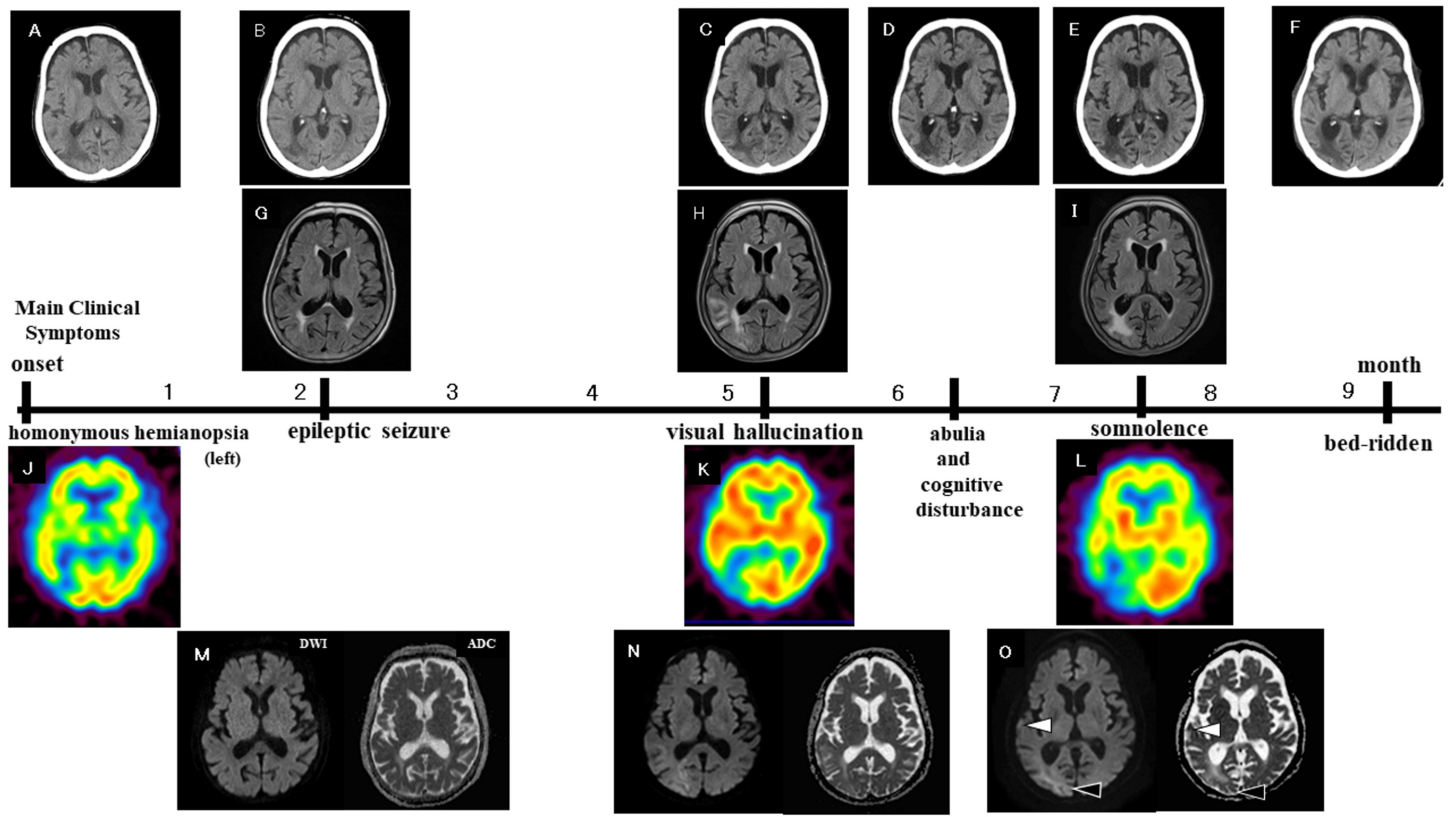
Main clinical symptoms and brain imaging findings in the first nine months after the initial onset CT (A-F): At the onset, WMLs were located within the right occipitotemporal lobe. At two months, WML had shrunk and was mostly confined to the right occipital lobe. By five months, WML had enlarged again within the right occipitotemporal lobe. From six months onward, the boundaries of the low-density areas in the right occipital lobe became more clearly defined. FLAIR (G-I): The high-intensity signal areas in the right occipitotemporal lobe were nearly identical to the low-density areas on CT. SPECT (J-L): At the onset, marked hypoperfusion was observed in the right occipital lobe and other cerebral lesions. Five months later, the hypoperfusion area extended from the right occipital lobe to the right temporal lobe. By seven months, the hypoperfusion in the right occipitotemporal lobe had further expanded, with hypoperfusion noted in additional regions except for the bilateral striatum and left occipital lobe. DWI/ADC (M-O): At two months, DWI showed iso-intensity signals across WML, and ADC showed no diffusion restriction in WML. At five months, DWI revealed a high-intensity signal area with unclear boundaries in WML, while ADC showed a mixture of non-diffusion-limited and diffusion-limited areas. By seven months, high-intensity signal areas with clear borders appeared in the occipital lobe (black arrowhead) and parts of the temporal lobe (white arrowhead) on DWI. These areas also exhibited diffusion limitations on ADC (black and white arrowheads). ADC: apparent diffusion coefficient map; DWI: diffusion-weighted image; FLAIR: T2-weighted fluid-attenuated inversion recovery; SPECT: ^123^I-IMP single photon emission CT perfusion brain image; WML: white matter lesion

Therefore, over the course of nine months from the onset of symptoms, both the clinical manifestations and imaging findings evolved or worsened, as illustrated in the diagram below (Figure 2).

At the onset of left homonymous hemianopia, cranial CT and 123I-IMP single-photon emission CT (SPECT) perfusion brain imaging were initially performed at the hospital. CT revealed low-density areas in the white matter of the right occipital lobe, extending into part of the right temporal lobe. SPECT demonstrated decreased blood flow that corresponded closely with the low-density areas observed on CT (Figure 2A, 2J).

Initially, the patient exhibited no cognitive impairment, and brain imaging did not reveal specific changes or findings characteristic of Alzheimer’s disease (not shown). The diagnosis at that time was a cerebral infarction in the right occipital lobe. Although hospitalization was recommended, the patient declined and opted for outpatient follow-up. At home, she gradually developed symptoms of left hemispatial neglect and an unsteady gait due to balance issues, occasionally bumping the left side of her body against doors or walls.

About two months after onset, the patient experienced generalized tonic-clonic seizures and was rushed to the emergency department. The EEG revealed spikes and sharp waves, and levetiracetam (1,000 mg) was administered intravenously to manage the seizures. CT showed a low-density area in the right occipital lobe. T2-weighted fluid-attenuated inversion recovery (FLAIR) imaging revealed a high-intensity signal localized around the dorsal horn of the right occipital lobe (Figure 2B, 2G). Diffusion-weighted imaging (DWI) showed no abnormal findings, and the apparent diffusion coefficient (ADC) map indicated no diffusion limitation in the same area where FLAIR showed the high-intensity signal (Figure 2M).

**Figure 3:**
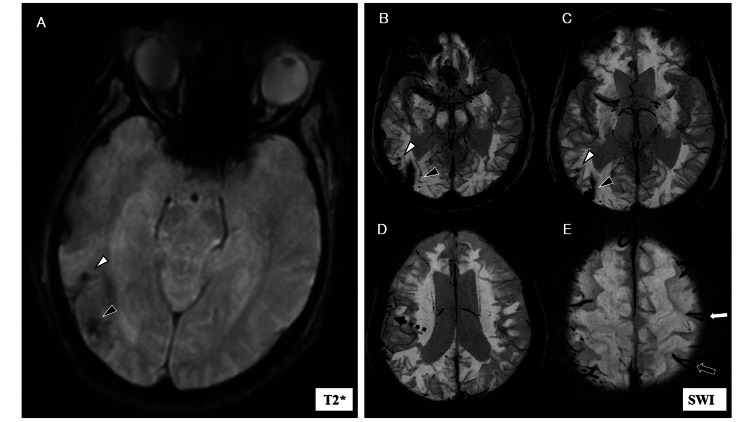
MRI T2* (at two months) and SWI images (at seven months) findings (A) At two months, T2* imaging revealed a CMB within the right temporal lobe (white arrowhead) and a low-signal focus in the border region of the right occipitotemporal lobe (black arrowhead). The low-signal focus was larger than the CMB and had an unclear boundary with the surrounding tissue. (B, C) At seven months, SWI images showed the CMB within the right temporal lobe (white arrowhead) and a fissured low-signal area extending into the deep white matter of the right occipital lobe (black arrowhead), surrounded by scattered CMBs. (D) A cluster of CMBs was observed in the cortical area of the right parietal lobe on SWI (dotted arrow). (E) Scattered CMBs were found in the right posterior parietal lobe, while in the left hemisphere, cSS was noted at the frontotemporal boundary region (white arrow) and the parieto-occipital boundary region (black arrow) on SWI. CMB: cerebral microbleed; cSS: cortical superficial siderosis; SWI: susceptibility-weighted imaging; T2*: T2*-weighted gradient-echo imaging

T2*-weighted gradient-echo imaging (T2*) revealed a CMB within the right temporal lobe, along with a pale, low-intensity signal lesion in the border region of the right occipitotemporal lobe. This lesion was larger and more irregular than the CMB, with an indistinct contour (Figure 3A). These T2* findings were re-evaluated using susceptibility-weighted imaging (SWI) seven months after onset (Figure 3B-3E).

Based on the T2* findings, CAA-ri was suspected as the underlying condition, and the patient was admitted on the same day. However, she strongly refused to undergo further examination and treatment and was subsequently followed up as an outpatient after one week. Although no recurrence of epileptic seizures was observed, visual hallucinations in the left homonymous hemianopia began to develop around the third month after onset. These hallucinations included unrealistic images, such as dwarfs attempting to take money from the patient’s wallet, as well as more realistic images, such as a salesperson operating a computer, all occurring within the left homonymous hemianopia.

In the fifth month following the onset of symptoms, the patient’s visual hallucinations intensified, and the boundary between hallucinations and real events often became blurred, occasionally leading to paranoia. This worsening of symptoms interfered significantly with her daily life and resulted in her admission to the psychiatric ward of the same hospital. Although the EEG did not show epileptiform discharges, it revealed slow waves around the right occipital lobe. CT imaging displayed WMLs extending from the right occipital lobe into the right temporal lobe, and FLAIR imaging also revealed a high-intensity signal in the same region (Figure 2C, 2H). DWI showed an indistinct high-signal intensity area, and the ADC map indicated a mix of diffusion-limited and non-diffusion-limited areas within the WML (Figure 2N). SWI imaging revealed clustered CMBs in the right parietal lobe and cSS in the left cerebral hemisphere (Figure 3B-3E).

SPECT imaging revealed an expansion of the area with reduced blood flow from the right occipital lobe into the temporal lobe, while other cerebral regions showed an overall increase in blood flow (Figure 2K). Blood tests conducted after admission indicated no inflammatory reactions or electrolyte abnormalities. CSF analysis showed normal levels of glucose, protein, and cell counts. Although corticosteroid therapy was initially considered, the patient’s prior chemotherapy history for hypopharyngeal cancer led to the decision to prioritize obtaining a definitive diagnosis through a brain biopsy. However, the patient refused to consent to the biopsy due to hallucinatory and delusional symptoms, irritability, and general refusal. Psychotropic medications, including tiapride hydrochloride (100 mg/day) and risperidone (2 mg/day), were prescribed to manage psychiatric symptoms, but no significant improvement was observed.

In the sixth month after onset, a noticeable decline in motivation became evident, accompanied by a gradual reduction in symptoms such as hallucinations, delusions, and irritability. Complaints of left homonymous hemianopia also diminished. During this period, cognitive function deteriorated rapidly, with the Mini-Mental State Examination (MMSE) score dropping from 25 in the fifth month to 15 in the sixth month. Motor function further declined, necessitating the use of a wheelchair for mobility.

CT imaging showed that WMLs were now confined to the right occipital lobe, and the low-density area had become more distinctly separated from the surrounding tissue over seven months (Figure 2D, 2E). Similarly, in FLAIR imaging, the contour of the high-intensity signal area within the right occipital lobe was more clearly defined (Figure 2I). DWI revealed a high-intensity signal area with well-defined borders in the right occipital lobe and part of the right temporal lobe, while the ADC map showed a diffusion-limited area corresponding to the high-intensity region observed on DWI (Figure 2O). SPECT imaging indicated a further expansion of the hypoperfusion area within the right temporal-occipital lobe, with hypoperfusion extending across nearly the entire cerebral area, except for the contralateral left occipital lobe and bilateral striatum (Figure 2L).

In the middle of the first seven months after onset, the patient required assistance with all daily activities and was transferred to our hospital due to significant cognitive and motor impairment, along with a tendency toward somnolence. Blood tests revealed no signs of inflammation or electrolyte abnormalities. CT imaging indicated that WMLs were confined to the right occipital lobe (not shown), but EEG results showed prominent slow waves. Although her general condition was stable, the physical examination revealed impaired balance and muscle weakness in both lower limbs, rendering her unstable when standing and necessitating wheelchair use for mobility.

Mentally, the patient no longer experienced hallucinations or delusions, but there was notable cognitive decline, with an MMSE score of 13 and a pronounced tendency toward somnolence. Given the progression of the illness up to the transfer and the symptoms and test results observed at our hospital, it was deemed likely that the patient was in an advanced stage of CAA-ri.

After an in-hospital discussion of the risks of disease progression and the benefits and potential harms of corticosteroid therapy, a decision was made to proceed with treatment. Following an explanation of the therapy to the patient and her husband, consent was obtained, and corticosteroid treatment was initiated in the latter half of the seventh month after the onset of the disease. The patient received high-dose steroid pulse therapy with methylprednisolone (1,000 mg/day intravenously for three days), followed by oral prednisolone (40 mg/day, 1 mg/kg) for one course, for a total of three courses administered.

By the end of the second course, there was some improvement in the tendency toward somnolence. However, the third course proved less effective, and no significant improvement was observed in CT findings (Figure 2F). Despite no complications from the corticosteroid treatment, the patient subsequently developed aspiration pneumonia due to worsening somnolence, and her overall condition gradually deteriorated. No further improvement was noted in her state of somnolence or overall condition, and she passed away from heart failure in the 11th month after the onset of symptoms.

## Discussion

CAA-ri is a disease associated with CAA, caused by an autoimmune reaction against Aβ that accumulates primarily in cerebral blood vessels [1,2,4]. Historically, destructive inflammation occurring transmural or intramural within the vessel wall where Aβ accumulates was classified as Aβ-associated vasculitis (ABRA), distinct from CAA-ri, which was characterized by perivascular inflammation. However, recent studies have shown that ABRA, like CAA-ri, is driven by an inflammatory response to Aβ deposition in cerebral blood vessels. Both conditions overlap and do not differ significantly in clinical presentation, imaging findings, or prognosis [2].

CAA-ri, within this context, presents with an acute to sub-acute clinical course, including symptoms such as cognitive decline, seizures, headaches, and focal neurological deficits. Brain imaging reveals characteristic cerebrovascular lesions, including asymmetric WMLs and CMBs. The diagnostic criteria proposed by Auriel et al., based on histological verification of these clinical symptoms and imaging findings, have a sensitivity of 82% and specificities of 97% and 68% for probable and possible cases of CAA-ri, respectively [4]. These criteria facilitate a highly accurate diagnosis and enable prompt treatment (Table 1).

**Table 1:** Criteria for the diagnosis of CAA-ri CAA-ri: cerebral amyloid angiopathy-related inflammation; CMB: cerebral microbleed; cSS: cortical superficial siderosis; ICH: intracerebral hemorrhage; WMH: white matter hyperintensity Source: Auriel et al. (2016) [4]

Probable CAA-ri	Possible CAA-ri
Age ≥40 years	Age ≥40 years
Presence of ≥1 of the following clinical features: headache, decreased consciousness, behavioral changes, focal neurological signs, or seizures, with the presentation not directly attributable to an acute ICH. MRI reveals unifocal or multifocal WMH that are asymmetric, extending into the immediately subcortical white matter, and this asymmetry is not due to previous ICH.	Presence of ≥1 of the following clinical features: headache, decreased consciousness, behavioral changes, focal neurological signs, or seizures, with the presentation not directly attributable to an acute ICH. MRI shows WMH extending to the immediately subcortical white matter.
Presence of ≥1 of the following corticosubcortical hemorrhagic lesions: cerebral macrobleed, CMB, or cSS	Presence of ≥1 of the following corticosubcortical hemorrhagic lesions: cerebral macrobleed, CMB, or cSS
Absence of neoplastic, infectious, or other causes	Absence of neoplastic, infectious, or other causes

However, since 37% of CAA-ri patients initially present with only a single symptom [5], early recognition of signs and symptoms and identification of characteristic findings, such as vasogenic edematous lesions in the cerebral white matter via MRI, can facilitate earlier diagnosis and more effective treatment. In this case, while recurrent epileptic seizures were not observed, the patient’s clinical condition worsened following the initial episode, leading to visual hallucinations, psychiatric symptoms, and rapid cognitive decline.

It has been observed that approximately 40% of CAA-ri patients experience at least one relapse after remission, and around 20% face multiple recurrences of symptoms [1]. Additionally, a study of 113 CAA-ri patients found that seizures occurring within three months of onset significantly reduced the likelihood of recovery [5]. Based on the data from this report and the clinical progression of the current case, it can be inferred that initiating treatment for CAA-ri approximately two months after the onset of left homonymous hemianopia, before the appearance of epileptic seizures, might have had a preventive effect on recurrence.

In addition to early and appropriate decision-making and treatment initiation, it is crucial to continue treatment for several months after remission and to carefully manage the reduction or discontinuation of therapeutic agents [1,2,5]. A notable symptom in this case was the occurrence of visual hallucinations within the left homonymous hemianopia. Since no seizure activity was observed on the EEG, these symptoms were considered non-epileptic complex hallucinations associated with the visual field loss.

Few reports address complex visual hallucinations in homonymous hemianopia. In 1985, Kölmel surveyed 120 patients with homonymous hemianopsia or quadrantanopia, investigating the presence, course, and nature of visual hallucinations, including whether they were still or moving images and the visual fields affected. This study identified 16 patients with complex visual hallucinations [8]. Notably, 13 of these patients were considered to have cerebrovascular disease, and in 14 cases, the hallucinations resolved completely. The involvement was primarily in the occipital lobe, although many cases also had lesions in the temporal and parietal lobes, with occasional edema in the temporal lobe. While the exact mechanism underlying complex visual hallucinations in homonymous hemianopia remains unclear, it is proposed that damage to the primary visual cortex in the occipital lobe may temporarily block external visual information, triggering internal information release. This process might involve visual image memory from the visual association areas in the parietal and temporal lobes [8]. A similar mechanism is suggested in Charles Bonnet syndrome, where visual hallucinations arise as a consequence of visual impairment [9,10].

In this case, complex visual hallucinations emerged approximately 10 weeks after the onset of homonymous hemianopia and resolved completely by 28 weeks after onset. At around five months, when the hallucinations were most active, imaging revealed vasogenic edema extending from the right occipital lobe to the right temporal lobe, with SPECT showing reduced blood flow in the same regions. Conversely, there was increased blood flow in the partially unaffected right temporal lobe and other cerebral areas. By seven months, when the hallucinations had disappeared, there was a significant reduction in blood flow across nearly all cerebral regions except for the left occipital lobe and bilateral striatum. This suggests that the hallucinations may have been due to overactivity in the visual association cortex and related areas compensating for blocked external visual information. The resolution of hallucinations could be attributed to damage in the visual association cortex and the broader visual information network, in addition to impairment in the primary visual cortex [9,10].

In contrast, it has been reported that patients with homonymous hemianopia often make saccadic eye movements toward the hemianopic field unconsciously to expand their visual field [11]. Additionally, Kölmel described a phenomenon where saccadic eye movements aimed at visual hallucinations within the hemianopic field could lead to their disappearance [8]. Therefore, it is possible that saccadic eye movements contributed to the resolution of visual hallucinations and the improvement in left homonymous hemianopia observed in this case. Although clear saccadic eye movements could not be confirmed during hospitalization, the increased blood flow in the left occipital lobe seen on SPECT after the hallucinations disappeared might be related to compensatory mechanisms associated with saccadic eye movements. Further studies, including investigations of eye movements, are needed to better understand these dynamics in patients with homonymous hemianopia and visual hallucinations.

In diagnosing CAA-ri, it is essential to differentiate it from other conditions that cause central nervous system inflammation and edema, such as infectious, neoplastic, and autoimmune diseases [2,4]. One critical differential diagnosis is reversible posterior leukoencephalopathy syndrome (PRES), which can be associated with anticancer drugs used in treating hypopharyngeal cancer [2,12]. PRES, first described by Hinchey et al. in 1996, is identified through clinical presentation and imaging findings [13]. Although PRES and CAA-ri share clinical similarities, such as angioedema predominantly in the occipital lobe, there are key differences. PRES often affects younger patients, with a mean age of 39 years in the original report, and typically presents with symmetrical and bilateral WMLs [13]. In contrast, CAA-ri is characterized by asymmetric WML and CMBs. The reversible nature of many clinical and imaging findings in PRES aligns with its association with malignant hypertension and renal failure, where the mechanism is believed to involve blood-brain barrier (BBB) disruption due to impaired vascular autoregulation from sudden blood pressure increases.

Conversely, in about 30% of cases, the BBB is disrupted by vascular endothelial damage caused by toxic factors from chemotherapeutic or immunosuppressive agents, leading to angiogenic edema in the cerebral white matter [12,14]. Cisplatin, a chemotherapeutic agent, is associated with PRES with an incidence of 2.7-10% [15]. While most angiogenic edema in the cerebral white matter is reversible, the cumulative dose of cisplatin required to trigger PRES remains unclear. The onset of PRES can occur anywhere from six hours to three months after starting treatment, necessitating vigilant monitoring throughout [15]. Additionally, hypertension and hypomagnesemia are common complications during cisplatin treatment. If PRES is suspected, managing blood pressure, maintaining adequate blood magnesium levels, and potentially reducing or discontinuing cisplatin are crucial measures [12,15].

In the present case, while the effect of cisplatin-induced cytotoxicity on the vascular endothelium cannot be ruled out, hypertension or hypomagnesemia were absent during treatment, and there was a 21-month gap between the end of cisplatin treatment and the onset of left homonymous hemianopia. This makes it highly unlikely that the symptoms were due to PRES. Nonetheless, recent studies using SWI have revealed a high incidence of CMBs in patients with PRES. In a study of 31 patients with PRES, with a mean age of 37.9 ± 19.1 years, CMBs were observed in approximately 60% of cases, suggesting a potential link between vasogenic edema and CMBs [16]. These findings indicate that PRES and CAA-ri may share similar mechanisms affecting small cerebral vessels, warranting further research into their pathogenesis and relationship.

Currently, there is no established treatment for CAA-ri. Management typically involves high-dose corticosteroids, such as intravenous methylprednisolone (1,000 mg/day for three to five days), followed by oral prednisone (1 mg/kg/day) for a certain period [2,3,5]. This regimen is estimated to improve clinical symptoms and imaging findings by approximately 70% and help prevent relapse [1]. Despite this, relapse occurs in about 40% of patients, necessitating prolonged treatment with careful tapering or discontinuation of medication [1]. A reduction in anti-Aβ42 antibodies in CSF after treatment may indicate treatment efficacy [3], although measuring these antibodies is not yet a common CSF test. The treatment may also have a diagnostic role in CAA-ri [17]. If corticosteroid therapy does not yield an effective response within three weeks, a brain biopsy or transition to an immunosuppressive agent, such as cyclophosphamide or azathioprine, may be considered [5,18].

In this case, three consecutive courses of corticosteroid therapy were administered. However, there are no established criteria for determining the optimal number of courses to assess treatment efficacy. Furthermore, uniform standards for the interval and duration of cyclophosphamide administration, a common immunosuppressive therapy, are currently lacking [1,2]. Given the high relapse rate associated with CAA-ri, there is a need for standardized guidelines for long-term treatment.

These findings and results from our report are based on a single case study and should not be generalized to CAA-ri as a whole. Further large-scale surveys and studies are recommended to obtain more accurate and reliable insights into CAA-ri.

## Conclusions

We reported the case of a female patient in her 70s with CAA-ri, initially suspected due to epileptic seizures, psychoneurotic symptoms, and left homonymous hemianopia, along with MRI findings of WMLs and CMBs in the right occipitotemporal lobe. The diagnosis was confirmed by a brain biopsy, which led to delayed treatment and an irreversible process. CAA-ri can be diagnosed with high accuracy based on characteristic clinical symptoms and imaging findings, and corticosteroid treatment is considered highly effective. Therefore, early diagnosis and timely initiation of treatment are crucial for managing CAA-ri.
